# Acute Toxicity and Hepatotoxicokinetic Studies of *Tamarindus indica* Extract

**DOI:** 10.3390/molecules16097415

**Published:** 2011-08-31

**Authors:** Uchechukwu U. Nwodo, Augustine A. Ngene, Aruh O. Anaga, Vincent N. Chigor, Igbinosa I. Henrietta, Anthony I. Okoh

**Affiliations:** 1 Applied and Environmental Microbiology Research Group, Department of Biochemistry and Microbiology, University of Fort Hare, Private Bag 1314, Alice, 5700 Eastern Cape, South Africa; E-Mails: vchigor@ufh.ac.za (V.N.C.); ladyisk@yahoo.com (I.I.H.); aokoh@ufh.ac.za (A.I.O.); 2 Department of Veterinary Medicine, University of Nigeria, Nsukka, 410002, Enugu State, Nigeria; E-Mail: austinearinze@yahoo.com (A.A.N.); 3 Department of Veterinary Physiology and Pharmacology, University of Nigeria, Nsukka, 410002, Enugu State, Nigeria; E-Mail: aruhanaga@yahoo.com (A.O.A.)

**Keywords:** brine shrimp, chicken embryos, hepatotoxicokinetics, fraction, liver enzymes

## Abstract

*Tamarindus indica* is widely used as a food and beverage and in traditional medicine. The apparent lack of dose standardization in herbal medicine necessitates the evaluation of the lethality *T*. *indica* on *Artemia salina* nauplii and chicken embryos via *in vitro* and *in vivo* techniques. Furthermore, hepatotoxicokinetics of the crude extract and fractions on Wister rats was also assessed. At concentrations of 200, 20 and 2 µg/mL, crude extract and fractions showed brine shrimp death percentages ranging from 86.70% to 3.30% and the sub-fractions showed death percentage ranges of 46.70% to 3.30%. Calculated LD_50_ values ranged from 832 µg/mL to 5,019 µg/mL. Dosing Wister rats with 25% and 50% concentration of LD_50_ determined for crude extract and fractions on chicken embryos showed an elevation in the ALT and AST levels in the serum. Brine shrimps and chicken embryos showed a positive correlation, with R^2^ values of 0.541 and 0.588 (P ≤ 0.05) for fractions and subfractions, respectively, as media for the lethality assay. Dose standardization in folk herbal medicine is imperative as *T*. *indica* used as food and medicine has been shown to be toxic at high doses. Brine shrimp and chicken embryos may be comparably used as medium for toxicity assay.

## 1. Introduction

Herbal medicine, a component of ethno-medicine, is as old as the history of man and spans all cultures [1,2]. The curative properties of herbs lie, incidentally, on secondary metabolites with *in situ* functions: toxins as deterrents to predation, pheromones attract insects for pollination, phytoalexins protect against bacterial and fungal attacks and allelochemicals which inhibit rival plants [3]. The development of pharmaceuticals favoured synthetic drugs and gave rise to the dominance of Western medicine; however, the observed toxicity of the latter and phenomenal emergence of strains resistant to antibiotics, for example, has warranted a return to or resurgence of herbal medicines in developed economies [4,5,6,7,8,9].

*Tamarindus indica*, an evergreen tree of the family *Leguminoseae*, has been used as both food and medicine, however it is often difficult to determine which use is more important [10,11,12]. Food uses include applications as a seasoning and as an ingredient in chutneys, curries, sauces, confections and beverages, as in some parts of the tropics, including Guatemala, Mexico and Puerto Rico, where commercially available beverage drinks, for example, *Tamarind ade* (trade name) have tamarind as the major ingredient [10]. As a medicine *T. indica* has been used as poultices for swollen joints, sprains and boils. Lotions made from it are used in treating conjunctivitis, as antiseptics, as vermifuges, treatments for dysentery, jaundice, erysipelas, hemorrhoids and various other ailments [12]. The bark of the tree is regarded as an effective astringent, tonic and febrifuge. Decoctions made from the fruit pulp is given as a remedy for indigestion, colic, gingivitis, asthma and eye inflammations; and lotions and poultices made from the bark are applied on open sores and caterpillar rashes. The powdered seeds are made into a paste for drawing boils and, with or without cumin seeds and palm sugar, are prescribed for chronic diarrhoea and dysentery [11,13,14]. *T. indica* has been empirically shown to have antibacterial activity [15,16].

Despite the numerous reports on the uses of *T. indica*, empirical documentation on its toxicity is scarce and those available do not account for its effects on hepatocytes, which is the primary target organ for drug metabolism, upon prolonged administration. This study is deemed necessary due to the vast range of applications of *T. indica* in folklore and mythical conceptions of herbal/natural products’ non toxicity [17]. Moreover, herbal medicine has additional limitations which includes; lack of standardization [18,19], inadequate knowledge on the role of all the active principles individually or in combination, and the implications of prolonged use [20,21]. Hence, the need for this study which aims at assessing acute toxicity of *T. indica* stem bark crude extract and fractions on *Artemia salina* nauplii (brine shrimps) and chicken embryos with subsequent evaluation of the hepatotoxic effects on Wister rats.

## 2. Results

### 2.1. Extraction and Fractionation of Extract

Column chromatographic fractionation of ethanol extract of *T. indica* stem bark yielded six fractions viz. TiA, TiB, TiC, TiD, TiE and TiF. TiA eluted first and TiF eluted last and each showed a single band on TLC, whereas TiB, TiC, TiD and TiE showed five, three, two and two bands, respectively. On refractionation of the fractions with multiple bands TiB yielded four distinct fractions, each expressing a single TLC band. These were designated B1, B2, B3 and B4, while TiC, TiD and TiE yielded two fractions each, that were similarly designated C1 and C2, D1 and D2, E1 and E2, respectively (Table 1). We have previously reported the phytoconstituents of fractions TiA–TiF, sub-fractions B1–B4; C1, C2; D1, D2; E1 and E2, and their antibacterial activities [15,16].

### 2.2. Brine Shrimp Lethality

Lethality assessment of *T. indica* crude extract and fractions on brine shrimps (nauplii) resulted in 70% (200 µg/mL) and 10% (20 µg/mL) nauplii death with crude extract (Table 2).

Similarly, at concentrations of 200, 20 and 2 (µg/mL), the range of percentages of lethality shown by the fractions TiA–TiF was 86.7% (TiE; 200 µg/mL) to 3.3% (TiC; 200 µg/mL). The calculated 50 percent lethal dose (LD_50_) for the crude extract is 1,542 µg/mL, while that of the fractions; TiA, TiB, TiC, TiD, TiE and TiF were 832, 2,753, 3,332, 1,626, 2,949 and 3,183 (µg/mL), respectively. Similarly, sub-fractions B1, B2, B3 and B4 showed 2,596, 3,980, 6,004 and 2,745 (µg/mL) as their respective 50% lethal dose (LD_50_) while C1 and C2 each yielded 3,183 µg/mL. Lastly, D1, D2, E1, and E2 showed 3,332, 1,717, 3,332, and 2,083 (µg/mL) as their respective LD_50_ values (Table 3).

### 2.3. Chicken Embryo Lethality

Alternative use of chicken embryos for toxicity assessment of *T. indica* crude ethanol extract and fractions via inoculation of 250, 25 and 2.5 (mg/mL) of crude extract and fractions into viable 10-day old pre-incubated embryonated chicken eggs with subsequent reincubation showed the following results: At 250 mg/mL for both crude ethanol extract and initial fractionation products (TiA, TiB, TiC, TiD, TiE and TiF), death percentages ranged 13.4 to 46.6, while at 25 mg/mL death percentages ranged from 34.4 to 13.4, respectively. The crude ethanol extract yielded an LD_50_ of 2,720, while TiA, TiB, TiC, TiD, TiE and TiF yielded 2,484, 5,019, 2,910, 4,344, 2,526 and 3,174, respectively (Table 4).

Similarly, the sub-fractions, at 250 mg/mL, killed chicken embryos with a percentage range of 6.6 to 40, while at 25 mg/mL, 6.6% to 33.4% deaths was observed. The calculated LD_50_s for these sub-fractions were; 2,720 (B1), 2,997 (B2), 4,483 (B3), 4,344 (B4), 3,312 (C1), 3,174 (C2), 3,132 (D1 and D2 each), 2,995 (E1) and 2,997 (E2), respectively (Table 5).

### 2.4. Correlation Analysis of Lethality Assay Media

Regression analysis of the LD_50_ values obtained with *T. indica* fractions TiA–TiF and subfractions B1–B4, C1, C2, D1, D2, E1 and E2 on brine shrimp nauplii and chicken embryos, for measurement of similarity as media for lethality assay, showed a positive correlation. The proximity matrix showed an association coefficient (R^2^ value) of 0.541 at P ≤ 0.05 against fractions TiA–TiF (Table 6), while sub-fractions; B1–B4, C1, C2, D1, D2, E1 and E2 similarly showed a linear association coefficient (R^2^ value) of 0.588 at P ≤ 0.05 (Table 7).

### 2.5. Hepatotoxicokinetics

Dosing Wister rats with *T. inica* crude extract and fractions at 25% and 50% concentrations of pre-determined LD_50_ on chicken embryos, showed an elevation in the AST and ALT levels in the serum. The ranges of AST levels after administering 25% concentration of extract and fractions were (U/L); 19.6 ± 3.2–35.7 ± 2.2 (crude extract), 23.6 ± 2.7–39.7 ± 1.9 (TiA), 24.5 ± 2.1–33.4 ± 2.7 (TiB), 18.2 ± 1.3–26.4 ± 3.5 (TiC), 23.6 ± 1.9–30.4 ± 3.3 (TiD), 22.6 ± 3.4–41.2 ± 2.7 (TiE) and 23.6 ± 1.1–31.9 ± 2.5 (TiF) respectively. Dose concentration increase to 50% LD_50_ resulted in further elevation in the serum AST levels (Table 8), with the following ranges (U/L); 36.2 ± 4.1–64.2 ± 2.2 (crude extract), 46.7 ± 5.1–69.2 ± 6.6 (TiA), 39.5 ± 3.0–63.0 ± 7.5 (TiB), 31.0 ± 2.5–57.4 ± 1.8 (TiC), 38.2 ± 3.8–57.1 ± 5.2 (TiD), 48.4 ± 3.1–80.0 ± 3.5 (TiE) and 39.6 ± 0.8–57.2 ± 0.5 (TiF) respectively. Similarly, dosing with 25% concentration of LD_50_ showed ranges of serum ALT levels as follows (U/L); 24.0 ± 1.1–37.5 ± 2.4 (crude extract), 28.4 ± 1.3–39.4 ± 2.7 (TiA), 25.4 ± 0.7–39.1± 1.4 (TiB), 18.7 ±2.1–35.3 ± 1.3 (TiC), 21.5 ± 2.5–38.3 ± 1.8 (TiD), 24.9 ± 1.4–39.2 ± 3.3 (TiE) and 22.7 ± 2.4–41.9 ± 1.6 (TiF) respectively. However, dose concentration increase to 50% LD_50_ similarly resulted in the elevation of serum ALT levels (Table 9). The ranges were (U/L); 46.9 ± 2.3–68.0 ± 1.3 (crude extract), 52.6 ± 3.3–71.2 ± 3.4 (TiA), 46.2 ± 2.3–68.9 ± 1.4 (TiB), 41.5 ± 1.7–61.5 ± 1.9 (TiC), 38.2 ± 2.6–67.8 ± 2.8 (TiD), 45.8 ± 1.2–61.9 ± 1.8 (TiE) and 39.5 ± 0.9–59.9 ± 1.7 (TiF) respectively. The peak of AST and ALT elevations at 25 and 50 (%) concentration were between the ranges of 12 to 18 days on the average. 

## 3. Discussion

### 3.1. Lethality Assays

The shrimp lethality assay proposed by Michael *et al.* [22] and later developed by Vanhaecke *et al.* [23] and Sleet and Brendel [24] is based on the ability of test agents to kill laboratory cultured *Artemia salina* nauplii (brine shrimp). The assay is considered a useful tool for preliminary assessment for toxicity [25], and it has been used for the detection of fungal toxins, plant extract toxicity or pharmacological activities and testing of dental material cytotoxicity, among others [26,27,28]. At 200 µg/mL concentration, TiE and TiA both killed 86.7% and 80.0% of the brine shrimps, which is higher than the result obtained with the crude extract (70%). This falls in line with the work of Dong *et al*. [29] where herbal extract purification increased toxicity. However, TiC, TiD and TiF showed results which were to the contrary. TiC showed the least toxicity even though it presented good bioactivity [15]. The results obtained in this research shows that herbal purification may yield products with higher or reduced toxicity when compared to the crude extract. In addition to high concentration playing major role in toxicity [30], the nature of bioactive constituent is similarly crucial as different compounds pose varied toxicity. Potency of bioactive compounds may not necessarily have a direct relationship with toxicity as we observed with fraction TiC. Chicken embryo toxicity showed result patterns somewhat similar to those obtained with brine shrimps.

### 3.2. Proximity Matrix Construct for Measure of Similarity between Assay Media

Proposed media for cytotoxicity assays include brine shrimps, chicken embryos, whole animals (guinea pigs and Wister rat) and plants. However, brine shrimp enjoys more usage as the assay is cheap and fast. In addition, the increased recognition of animal rights will continue to place brine shrimp as an indispensable tool for toxicity testing. A chicken embryo has not enjoyed the same status as brine shrimps, even though it is proposed as a similar tool. However, lethality testing using both assay media and analyzing the results obtained thereof, for measure of similarity between the assay medium indicated a positive correlation. The use of brine shrimps and chicken embryos showed 60% similarity; consequently this result may be interpreted to mean that chicken embryo could serve in the determination of LD_50_, but not in place of the brine shrimps and *vice versa* as the variation in sensitivity is large.

### 3.3. Hepatotoxicokinetics

Dosing Wister rats with 25% and 50% LD_50_ concentrations of crude extract and fractions showed a time dependent elevation in liver enzymes (ALT and AST) in the serum. At 25% LD_50_ concentration, serum ALT steadily increased over time and peaked at day 12 for TiA, TiD and TiF. Others showed peak values at 15 and 18 days, respectively. At an increased dose (50% LD_50_), a different pattern was observed as some fractions showed peak ALT values at different days from the former: crude extract (day 15), TiA (day 18) and TiC (day 12). Serum AST levels followed a similar pattern as ALT, thus showing variations in peak time with concentration for different fractions. The elevations in serum ALT and AST levels are associated with hepatocellular injury [31], and these were observed with Wister rats when dosed with sub-lethal doses (25% and 50%) of *T*. *indica*. However, only 50% LD_50_ concentrations were able to elicit serum liver enzymes with values above the normal threshold for healthy hepatocytes (AST; 5–34 U/L and ALT; 7–56 U/L). ALT and AST are released from damaged hepatocytes into the blood following hepatocellular injury or death, although they can originate from other tissues. However, elevation of ALT and AST that is the five times higher than the normal and over range has been used as a paradigm in the classifications of liver disease states [31]. The AST and ALT levels observed in this study are nowhere near the values that signify a disease stated, but this gives an insight on what may occur if a lethal dose of *T. indica* is administered to the experimental animals. The patterns to which the liver enzymes peak over time with subsequent stabilization or decline may suggest acclimatization to the toxicants.

## 4. Experimental Section

### 4.1. Plant Materials

The stem bark of the study plant was obtained from the woods of More in Sokoto South Local Government Area, Sokoto State, Nigeria. The plant was identified taxonomically and a voucher specimen deposited at the Herbarium of the Department of Botany, University of Nigeria, Nsukka.

### 4.2. Extractions of the Plant Materials

Fresh stem bark of *T. indica* were rinsed thoroughly in running tap water, chopped to tiny pieces and air dried at room temperature (~27 °C). The dried stem bark was pulverised and 50 g of the milled material was macerated in absolute ethanol (Sigma-Aldrich, 200 mL) and left to stand for about 4 h. The preparation was filtered through Whatman No. 1 filter paper and the filtrate concentrated to dryness under a steady air current. The extract was stored in sterile containers at a temperature of 4 °C until use.

### 4.3. Fractionation of Extract

The ethanol extract of the stem bark of *T. indica* were re-constituted in absolute ethanol and spotted on analytical TLC plates (silica gel G_600_; 0.25 mm thickness) and ethanol/ethyl acetate 6:4 systems showed better separation of the spotted extract. Fractionation and re-fractionation of the extracts were carried out using standard methods [15].

### 4.4. Brine Shrimp Lethality Assay

The brine shrimp lethality bioassay technique was as described by Meyer *et al*. [32] and McLaughlin *et al*. [27]. The *Artemia salina* (Leach) eggs (Interpet Ltd., Dorking, UK) were obtained from the Veterinary Pharmacology Department, University of Nigeria, Nsukka. To natural seawater obtained from the Atlantic Ocean at Bar Beach Lagos, Nigeria (about 200 mL), *Artemia salina* eggs (20 mg) were added and incubated at room temperature (~27 °C) for about 48 h in a hatchery. Brine shrimp larvae (nauplii) were separated from the eggs by picking with a Pasteur pipette into small beakers containing seawater. Three sets of 10 *Artemia salina* nauplii were, each, exposed to 200, 20 and 2 µg/mL of crude stem bark extract and its fractions in sea water. This was done in triplicate and the shrimp were then incubated at room temperature (~27 °C) for about 24 h after which surviving shrimps of each treatment was recorded and the lethal dose that killed 50% of the shrimps (LD_50_) determined by regression analysis.

### 4.5. Chicken Embryo for Lethality Assay

Embryonated chicken eggs (53 ± 0.6 g) were obtained from the intensive reared poultry of the Virology Unit, Department of Microbiology, University of Nigeria, Nsukka. Egg incubation was within five days of lay, in a humidified incubator at 37 °C for 9–10 days with candling and manual turning, thrice daily. Unfertilized eggs and eggs with dead embryos were discarded.

### 4.6. Chicken Embryo Lethality Assay

Three sets of five ten-day old embryonated chicken eggs were, each, inoculated with a single dose (100 µL) of 250, 125 or 62.5 mg/mL of the crude stem bark extract following standard procedures [33]. The eggs were incubated in a humidified incubator, turned thrice daily and candled once every 24 h to assess the viability of the chick embryo; eggs were discarded if embryo death occurred within 24 h [34] and beyond, death was recorded. Similar procedure was repeated with all the chromatographic fractions obtained. The control groups were inoculated with sterile distilled water and incubated in the same conditions as the treatment groups.

### 4.7. Animal Study

Sixty-four healthy male Wister rats aged three weeks were purchased from the Animal Unit, Department of Veterinary Parasitology, University of Nigeria, Nsukka. The rats were reared in metabolic cages at room temperature (~27 °C), relative humidity 47 ± 5% and 12 h light/dark cycle at the animal house, Virology Unit, Department of Microbiology, University of Nigeria, Nsukka. Commercial pellet feed (Guinea feeds) and water was available *ad libitum*. After five weeks of breeding, each rat weighed 150–180 g on the average. The rats were randomly placed into 16 homogenous weight groups with four rats in each group. The first seven groups were dosed with 25% LD_50_ concentration (LD_50_ pre-determined on chicken embryo; Section 2.6) of crude and the six fractions (TiA–TiF) while the second seven groups were similarly dosed with 50% LD_50_ and the controls received distilled water. At 25% LD_50_, dose translates to the following concentrations (mg/mL); 680 (crude extract), 621(TiA), 1,255 (TiB), 730 (TiC), 1,086 (TiD), 632 (TiE) and 794 (TiF) while 50% LD_50_ similarly translates to (mg/mL); 1,360 (crude extract), 1,242 (TiA), 2,510 (TiB), 1,460 (TiC), 2,172 (TiD), 1,263 (TiE) and 1,587 (TiF), respectively. The solvent for reconstitution was sterile distilled water and crude extract and fractions (200 µL) were administered orally per 20 g of body weight routinely at 24 h intervals for 21 days. At 72 h (three days) interval the rats were bled via ocular puncture, and blood samples were allowed to clot and serum separated by centrifugation (3,000 rpm; 5 min). Animal studies where in compliance to the ethical procedure of the Animal Use and Care Committee, Faculty of Veterinary Medicine, University of Nigeria, Nsukka which corresponds with NIH guidelines [35].

### 4.8. Liver Enzyme Assay-Aspartate Aminotransferase and Alanine Aminotransferase

Aspartate aminotransferase (AST) levels in each serum sample were determined using AST reagent kits (Ouimica Clinica Aplicada, Amposta, Spain). The AST reagents consisted of phosphate buffer (100 mM) pH 7.4, α-ketoglutaric acid (2 mM) and L-aspartic acid (100 mM) contained in 100 mL of substrate solution. Other reagents used include a colour developer (1 mM of 2,4-dinitrophenyl-hydrazine [DNPH]) and 4N NaOH which was diluted (1/10) with deionised water). The assays were conducted according to the manufacturers’ instruction protocol. Thus, AST substrate (500 µL) was pipetted into a test tube and incubated in a water bath for 5 min at 37 °C after which serum (100 µL) was added with further incubated for 30 min at the same temperature. Afterwards, DNPH (500 µL) was added and held at room temperature (~27) for 20 min followed by addition of dilute NaOH (0.4 N, 5000 µL) with further incubation for 15 min. The absorbance as measured by optical density (OD) was read using a colorimeter (EI, Yong Jing, China) at 505 nm wavelength against blank. The ALT reagent was likewise composed of phosphate buffer, pH 7.4 (100 mL), α-ketoglutaric acid (2 mM) and L-alanine (100 mM) in 100 mL of substrate solution. The same colour developer, NaOH (0.4 N) and assay protocol was used as in AST assay.

### 4.9. Statistical Analysis

Brine shrimp and chicken embryo lethality was calculated using Probit analysis, while analysis of variance (ANOVA) was used to analyze aspartate aminotransferase and alanine aminotransferase levels obtained in the hepatotoxic assay on Wister rats. The statistic package used for the analysis is SPSS for windows version 17 (SPSS Inc., Chicago, IL, USA).

## 5. Conclusions

This study shows that brine shrimps and chicken embryos are not comparable media in cytotoxicity assays and the variation in sensitivity may be attributed to genetic variability among other factors. However, the positive association obtained between these assay media implies that both media could be used in cytotoxicity testing of bioactive compounds. The assertion that “the dose makes the poison” holds true as *T. indica*, the plant, with vast applications in food, beverages, and folk medicine, among others, has been shown to be intrinsically hazardous, at high doses, like all chemical agents. The abnormal serum liver chemistry obtained in this study has shown some insight into the mechanism of toxicity of *T. indica*, this is necessary because there is a paucity of information on herbal toxicity and more so their effects on the hepatocytes which is where detoxification and drug metabolism, among other processes, take place. Hence, toxic responses obtained with laboratory animals will predicate responses in humans at high doses over time.

## Figures and Tables

**Table 1:** Chromatographic separations of the stem bark of *Tamarindus indica*.

	Products of first fractionation	Tracks on TLC	Refractionation products	Tracks on TLC
Crude EOH extract of stem bark	TiA TiB TiC TiD TiE TiF	1 5 3 2 2 1	- B1, B2, B3, and B4 C1 and C2 D1 and D2 E1 and E2 -	- 1 1 1 1 -

**Table 21:** Lethality of *T. indica* fractions on Brine shrimp.

Fractions	Concentrations/Death rate (%)			
	200 ppm	20 ppm	2 ppm	LD_50_
Crude	70.0	10.0	0.0	1542
TiA	80.0	46.70	13.30	832
TiB	26.70	3.30	0.0	2753
TiC	3.30	0.0	0.0	3332
TiD	23.30	3.30	0.0	2950
TiE	86.70	0.0	0.0	1626
TiF	23.30	6.70	0.0	3183

**Table 3:** Lethality of *T. indica* sub-fractions on Brine shrimp.

Sub-fractions	Concentrations/Death rate (%)			
	200 ppm	20 ppm	2 ppm	LD_50_
B1	30.0	3.30	0.0	2596
B2	16.70	6.70	0.0	3980
B3	10.0	6.70	0.0	6004
B4	10.0	0.0	0.0	2745
C1	3.30	0.0	0.0	3332
C2	3.30	0.0	0.0	3332
D1	23.30	6.70	0.0	3183
D2	3.30	0.0	0.0	3332
E1	60.0	13.30	0.0	1717
E2	46.70	13.30	0.0	2083

**Table 4:** Lethality of *T. indica* fractions on chicken embryo.

Sub-fractions	Concentrations/Death rate (%)			
	250 mg/mL	25 mg/mL	2.50 mg/mL	LD_50_
Crude	40.0	13.40	0.0	2720
TiA	46.60	33.40	6.60	2484
TiB	13.40	13.40	0.0	5019
TiC	13.40	0.0	0.0	2920
TiD	13.40	6.60	0.0	4344
TiE	46.60	0.0	0.0	2526
TiF	26.60	6.60	0.0	3174

**Table 5:** Lethality of *T. indica* sub-fractions on chicken embryo.

Sub-fractions	Concentrations/Death rate (%)			
	250 mg/mL	25 mg/mL	2.50 mg/mL	LD_50_
B1	40.0	13.40	0.0	2720
B2	33.40	26.67	0.0	2997
B3	33.40	26.60	20.0	4483
B4	33.40	6.60	0.0	4344
C1	26.60	13.40	0.0	3312
C2	26.60	6.60	0.0	3174
D1	6.60	0.0	0.0	3132
D2	6.60	0.0	0.0	3132
E1	33.40	33.40	0.0	2995
E2	33.40	26.60	0.0	2997

**Table 6:** Proximity matrix of *Artemia salina* nauplii and chicken embryos calculated from LD_50_ yield of *T. indica* fractions.

Tool used to determine LD_50_	Correlation between vectors of values	
	*Artemia salina* nauplii	Embryonated chicken eggs
*Artemia salina* nauplii Embryonated chicken eggs	1.000 0.541	0.541 1.000

P = 0.05.

**Table 7:** Proximity matrix of *Artemia salina* nauplii and chicken embryos calculated from LD_50_ yield of *T. indica* refractionations.

Tool used to determine LD_50_	Correlation between vectors of values	
	*Artemia salina* nauplii	Embryonated chicken eggs
*Artemia salina* nauplii Embryonated chicken eggs	1.000 0.588	0.588 1.000

P = 0.05.

**Table 8:** Aspartate aminotransferase concentration at *T*. *indica* dosage over time.

Days	Crude	TiA	TiB	TiC	TiD	TiE	TiF	Control
	25% Concentration of Extract and fractions (mg/20 g body weight)/AST levels (U/L)							
3	19.6 ± 3.2	23.6 ± 2.7	24.5 ± 2.1	18.2 ± 1.3	23.6 ± 1.9	22.6 ± 3.4	23.0 ± 1.1	17.3 ± 1.7
6	24.5 ± 4.6	29.1 ± 4.4	26.9 ± 3.1	20.8 ± 2.3	25.6 ± 2.1	29.1 ± 1.9	25.0 ± 1.8	19.6 ± 2.0
9	27.6 ± 6.2	33.5 ± 6.3	31.8 ± 1.8	22.2 ± 1.9	28.7 ± 3.9	36.5 ± 2.0	29.4 ± 1.9	19.9 ± 1.5
12	33.5 ± 2.9	39.7 ± 1.9	32.6 ± 1.7	25.0 ± 3.1	30.4 ± 3.3	40.5 ± 3.9	31.9 ± 2.5	20.2 ± 1.9
15	34.8 ± 1.8	39.1 ± 3.1	33.3 ± 2.4	26.2 ± 2.2	29.4 ± 1.5	41.2 ± 2.7	31.4 ± 1.9	18.9 ± 1.7
18	35.7 ± 2.2	37.8 ± 2.0	33.3 ± 1.6	26.4 ± 3.5	29.1 ± 0.9	40.8 ± 1.7	30.9 ± 1.4	18.2 ± 2.1
21	35.0 ± 3.1	37.4 ± 2.8	33.4± 2.7	25.9 ± 2.0	28.3 ± 1.1	38.9 ± 1.3	30.2 ± 0.9	18.7 ± 1.8
	**50% Concentration of Extract and fractions (mg/20 g body weight)/AST levels (U/L)**							
3	36.2 ± 4.1	46.7± 5.1	39.5 ±3.0	31.0 ± 2.5	38.2 ± 3.8	48.4 ± 3.1	39.6 ± 0.8	17.3 ± 1.7
6	41.7 ± 3.9	52.3 ± 7.2	49.1 ± 4.4	48.6 ± 3.1	48.7 ± 2.9	53.9 ± 3.7	43.7 ± 2.3	19.6 ± 2.0
9	58.9 ± 5.2	57.8 ± 4.2	57.5 ± 2.9	53.2 ± 2.7	54.5 ± 1.9	65.0 ± 2.7	49.7 ± 1.9	19.9 ± 1.5
12	61.6 ± 3.1	62.1 ± 8.1	63.0 ± 7.6	57.4 ± 1.8	57.1 ± 5.2	71.2 ± 4.2	57.2 ± 0.5	16.8 ± 1.9
15	64.2 ± 2.2	68.3 ± 5.3	58.3 ± 4.9	46.9 ± 4.1	53.1 ± 5.3	77.6 ± 5.2	51.4 ± 2.6	18.9 ± 1.7
18	61.9 ± 1.9	69.2 ± 6.6	55.8 ± 2.5	42.5 ± 2.2	49.3 ± 3.3	80.0 ± 3.5	45.2 ± 3.2	16.8 ± 2.1
21	59.7 ± 1.3	67.2 ± 5.9	54.6 ± 1.8	38.9 ± 3.9	44.6 ± 2.5	80.0 ± 2.2	41.5 ± 1.8	19.5 ± 1.8

Normal range of AST value for healthy animals 5–34 U/L.

**Table 9:** Alanine aminotransferase concentration at *T*. *indica* dosage over time.

Days	Crude	TiA	TiB	TiC	TiD	TiE	TiF	Control
	**25% Concentration of Extract and fractions (mg/20g body weight)/ALT levels (U/L)**							
3	24.0 ± 1.1	28.4 ± 1.3	25.4 ± 0.7	18.7 ± 2.1	21.5 ± 2.5	24.9 ± 1.4	22.7 ± 2.4	18.6 ± 1.3
6	27.1 ± 1.7	32.5 ± 2.6	26.9 ± 1.1	21.6 ± 1.6	27.2 ± 1.2	29.4 ± 1.7	25.2 ± 1.5	20.3 ± 2.7
9	31.9 ± 2.3	37.2 ± 1.8	33.2 ± 2.3	29.6 ± 1.4	38.3 ± 1.8	37.1 ± 1.8	33.7 ± 1.7	19.7 ± 1.8
12	34.6 ± 1.7	39.4 ± 2.7	39.1 ± 1.4	35.1 ± 2.2	35.4 ± 2.4	39.2 ± 3.3	41.9 ± 1.6	19.2 ± 1.5
15	37.5 ± 2.4	36.0 ± 3.5	38.0 ± 3.1	35.3 ± 1.3	29.6 ± 1.6	36.1 ± 1.7	35.9 ± 2.4	19.2 ± 1.7
18	35.1 ± 1.2	34.7 ± 1.8	35.2 ± 1.2	33.1 ± 2.7	24.6 ± 1.1	30.2 ± 1.6	32.7 ± 3.1	20.3 ± 1.9
21	34.9 ± 1.9	32.9 ± 2.3	30.3 ± 1.5	32.2 ± 1.3	24.1 ± 2.8	27.6 ± 1.5	30.5 ± 1.9	19.5 ± 1.5
	**50% Concentration of Extract and fractions (mg/20g body weight)/ALT levels (U/L)**							
3	46.9 ± 2.3	52.6 ± 3.3	46.2 ± 2.3	41.5 ± 1.7	38.2 ± 2.6	45.8 ± 1.2	39.5 ± 0.9	15.9 ± 1.3
6	58.2 ± 1.3	59.1 ± 2.7	53.1 ± 1.9	47.2 ± 0.9	46.7 ± 2.3	51.4 ± 1.5	43.8 ± 1.3	21.0 ± 2.7
9	62.4 ± 2.5	67.4 ± 1.5	61.8 ± 3.1	53.8 ± 2.1	58.2 ± 1.9	56.6 ± 2.6	59.9 ± 1.7	19.7 ± 1.8
12	67.1 ± 2.2	71.2 ± 3.4	63.4 ± 2.8	57.9 ± 2.6	62.3 ± 3.1	61.9 ± 1.8	57.3 ± 2.8	19.2 ± 1.5
15	68.0 ± 1.3	68.1 ± 2.6	68.9 ± 1.4	61.5 ± 1.9	67.8 ± 2.8	58.8 ± 2.1	54.1 ± 3.5	18.5 ± 1.7
18	65.9 ± 1.5	66.3 ± 1.2	65.2 ± 1.7	54.7 ± 2.1	57.2 ± 1.9	50.9 ± 2.2	50.2 ± 2.3	23.0 ± 1.9
21	66.3 ± 1.3	62.5 ± 2.7	60.9 ± 2.3	51.9 ± 1.5	54.1 ± 1.7	55.9 ± 1.4	43.2 ± 2.7	19.5 ± 1.5

Normal range of ALT value for healthy animals 7–56 U/L.
